# Four-month course of adjuvant dabrafenib in patients with surgically resected stage IIIC melanoma characterized by a BRAFV600E/K mutation

**DOI:** 10.18632/oncotarget.21072

**Published:** 2017-09-16

**Authors:** Parisa Momtaz, James J. Harding, Charlotte Ariyan, Daniel G. Coit, Taha Merghoub, Billel Gasmi, Daoqi You, Agnes Viale, Katherine S. Panageas, Aliaksandra Samoila, Michael A. Postow, Jedd D. Wolchok, Paul B. Chapman

**Affiliations:** ^1^ Memorial Sloan Kettering Cancer Center, New York, New York, USA; ^2^ Weill Cornell Medical College, New York, New York, USA

**Keywords:** cfDNA, digital PCR, drug cost, relapse-free survival

## Abstract

**Background:**

We tested the hypothesis that a 4-month course of adjuvant dabrafenib in stage IIIC BRAF-mutated melanoma would improve 2 year RFS from 24% to 51%, and that tumor-derived cell free DNA (cfDNA) in plasma would correlate with and predict recurrence.

**Methods:**

Patients with stage IIIC BRAF V600E/K mutated melanoma who were free of disease after surgical resection received 4 months of adjuvant dabrafenib. Patients were evaluated with imaging at baseline, at the end of cycles 2, 4, 6, then every 3 months until disease relapse or 2 years, whichever came first. Serial blood samples were collected for evaluation of cfDNA at the same time.

**Results:**

21/23 patients enrolled were evaluable; 2 patients withdrew consent during the first week of treatment. The 2 year RFS was 28.6% (95% CI 12-48%). The estimated overall survival at 2 years was 78% (95% CI 51-91%). cfDNA detection had a 53% sensitivity in relapsing patients but cfDNA detection did not provide lead-time advantage over CT scanning.

**Conclusion:**

A 4-month course of adjuvant dabrafenib did not result in a detectable improvement in 2-year RFS. cfDNA was less sensitive than standard CT imaging and did not provide a lead-time advantage in detecting relapse.

## INTRODUCTION

Treatment of patients with metastatic BRAF V600E/K-mutated melanoma with the FDA approved RAF inhibitors, vemurafenib and dabrafenib, leads to rapid tumor shrinkage in most patients. This has translated into improvement in progression free survival (PFS) and a modest improvement in OS compared to dacarbazine chemotherapy in the case of vemurafenib [1, 2]. The majority of major responses occur by 6 weeks, the time of the first radiographic evaluation [3–5]. This indicates that RAF inhibitors mediate substantial tumor cell death within two months. Late responses are uncommon and most tumors develop resistance to RAF inhibition after a median of 7 months. The addition of MEK inhibitors to the RAF inhibitors results in a modest improvement in PFS and OS and is now a standard of care for patients with BRAF mutated metastatic melanoma, although combination therapy with dabrafenib and trametinib can be associated with increased rates of fevers/chills.

Since the number of tumor cells in the adjuvant setting is many orders of magnitude lower than in the metastatic setting and given the magnitude of cell kill in the first two months, we hypothesized that a short course (4 months) of adjuvant dabrafenib would be sufficient to eradicate remaining melanoma cells and improve relapse-free survival (RFS) significantly.

We previously reported that in our institutional database, surgically-resected stage IIIC melanoma patients had a 24% RFS at 2 years from the time of surgery [6]. This cohort consisted of 280 patients (65% male) with a median age of 56.5 years. The site of the initial primary melanoma was extremity (50%), trunk (26%), head/neck (15%), or unknown (9%). Here we evaluated the benefit of a 4-month course of adjuvant dabrafenib in surgically resected stage IIIC patients to see if treatment would improve 2-year RFS from 24% to 51%. We did not include a MEK inhibitor for two reasons: at the time we conducted this trial, the addition of MEK inhibitor had been shown to improve RFS only minimally and there were no OS data [7]. Second, given the uncertain benefit of adding a MEK inhibitor, we wanted to avoid the increased toxicities of fever, chills, and constitutional symptoms. This trial was not designed to give a definitive answer as to the efficacy of adjuvant RAF inhibition but rather to determine if there is a signal worth pursuing in future randomized trials from a relatively short course of RAF inhibition. In addition, we obtained serial peripheral blood samples from all patients on this trial to measure cell free DNA (cfDNA) levels to evaluate the hypothesis that quantitative changes in cfDNA will be able to detect melanoma recurrence with the hope that cfDNA might replace standard CT scan imaging for detection of relapse.

## RESULTS

### Patients

Between 11/2012 and 12/2015, 23 patients with stage IIIC BRAF V600E/K mutated melanoma provided written informed consent and enrolled in the study. Of the 23 patients, 21 were evaluable. Two patients withdrew consent after having received approximately 4 days of dabrafenib and are considered for toxicity evaluation only. Of the 21 evaluable patients (Table 1), 15 were men and 6 women; the median age was 54 (range 18-76 years old). 17 patients had a BRAF V600E mutation and 4 patients had a BRAF V600K mutation. The median number of days from surgical resection to dabrafenib start was 42 days (range 25-81 days). Pathologic characteristics of the primary tumor were notable for presence of ulceration in 9 patients (42%), macroscopic lymph nodes in 14 patients (67%), and extranodal extension in 5 patients (24%). 76% of patients had N3 disease.

**Table 1:** Patient and Disease Characteristics

	N = 21
Sex	
Male	15 (71%)
Female	6 (29%)
Median Age (years)	54 (range 18-76)
Mutation	
BRAF V600E	17 (81%)
BRAF V600K	4 (19%)
Median days from surgery to dabrafenib start	42 (range 25-90)
Ulceration	
Present	9 (42%)
Absent	6 (29%)
Unknown	6 (29%)
Pathologic Staging IIIC^**^	
T(any)b N1b	3 (14%)
T(any)b N2b	1 (5%)
T(any)b N2c	1 (5%)
Any T N3	16 (76%)
LN involvement	
Microscopic	7 (33%)
Macroscopic	14 (67%)
Site of Primary Melanoma	
Trunk	8 (38%)
Upper Extremity	1 (5%)
Lower Extremity	4 (19%)
Acral	5 (24%)
Head/Neck	2 (9%)
Unknown	1 (5%)
Post-relapse systemic therapy	
RAFi +/− MEKi	6 (29%)
Checkpoint Inhibitors	15 (71%)

^*^LN = lymph node; ^**^AJCC v.7 Staging.

### Clinical outcomes

Most patients (17/21; 81%) completed 4 months of adjuvant dabrafenib. Of the 4 patients who did not complete 4 cycles, 3 patients came off treatment after 3 cycles due to drug related toxicity; another patient came off treatment after 2 cycles due to the diagnosis of a second primary melanoma.

**Figure 1:**
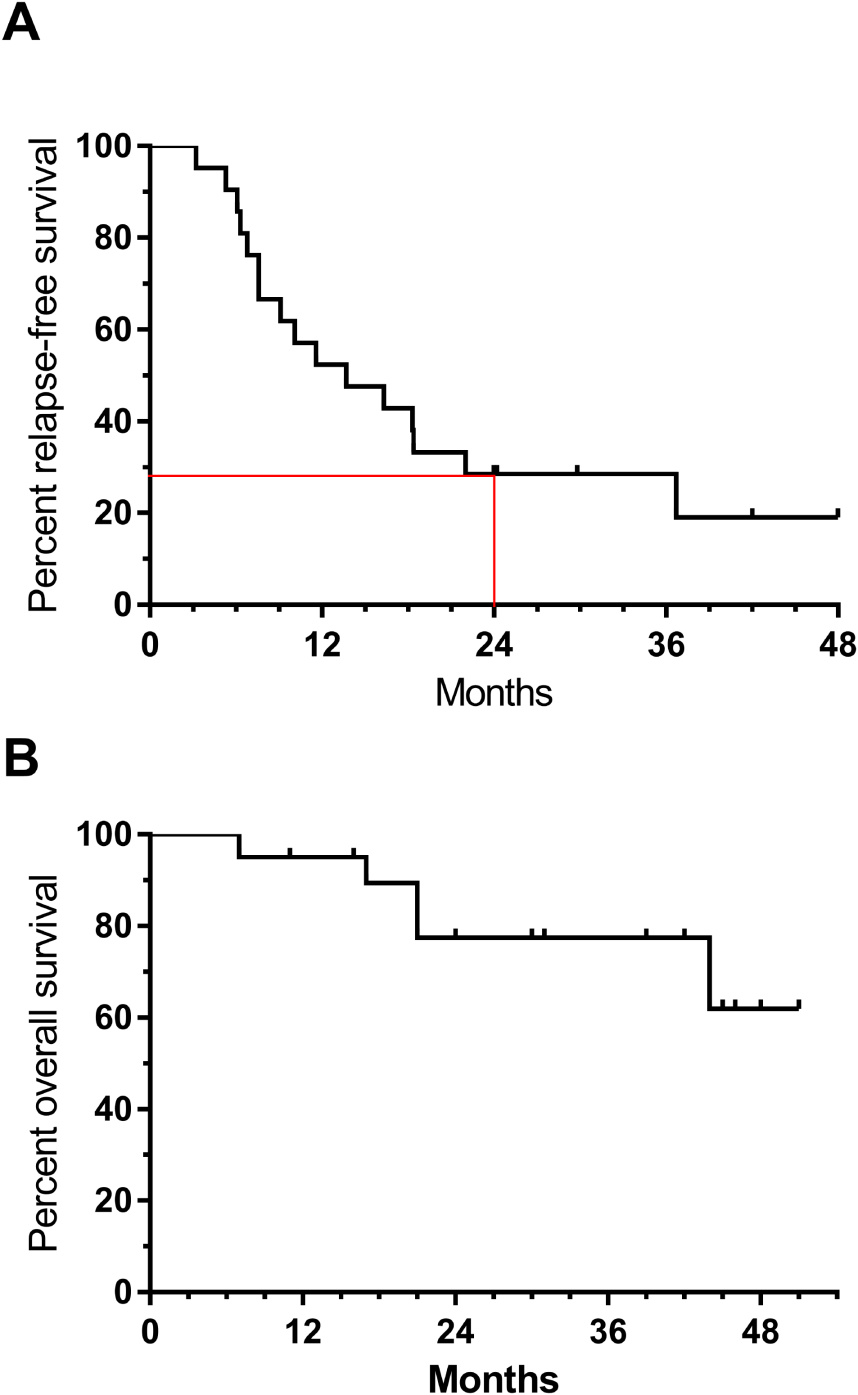
Relapse-free **(A)** and overall survival **(B)** in patients treated with adjuvant dabrafenib. Tick marks indicate censored patients. Red lines indicate 2-year RFS.

Despite adjuvant dabrafenib, most patients (15/21; 71%) relapsed before 24 months; one patient relapsed after 24 months. We noted that 5 of the patients relapsed in the brain; in 3 of these patients brain was the only site of relapse. Five patients have not yet relapsed and have been followed for 24-48 months. The 2 year RFS from the date of surgery for all patients was 28.6% (95% CI 12, 48%) (Figure 1A). This result did not meet the pre-specified criteria for rejecting the null hypothesis meaning we were not able to detect improvement in 2 year RFS. Of note, the estimated overall survival at 2 years was 78% (95% CI 51, 91%). The median OS has not yet been reached (Figure 1B).

One question was whether adjuvant dabrafenib therapy would result in resistance to subsequent RAF inhibition in the metastatic setting among patients who relapsed. Following relapse of melanoma, 6 of our patients received either single agent RAF inhibitor or combination RAF/MEK inhibitor therapy. The median time from the last dose of adjuvant dabrafenib to RAF inhibition treatment in the metastatic setting was 8 months (range 4-20 months). Five patients had a clinical and/or radiographic response to therapy. Median time on RAF inhibition in the metastatic setting was 5 months (range 1-22 months). This suggests that relapsing tumors remained sensitive to RAF inhibition. [8]

More frequently, patients received checkpoint inhibitors at relapse. After relapse, 15 patients received immunotherapy with checkpoint inhibitors. Five patients were treated initially with ipilimumab; 3 of these patients went on to receive anti-PD1 antibody therapy. Five patients were treated initially with anti-PD-1 antibody one of whom went on to receive ipilimumab. Five other patients were treated with upfront ipilimumab/nivolumab combination. Eight patients have had an objective response to immunotherapy and are on active surveillance; two patients remain on immunotherapy.

### Toxicity

All 23 enrolled patients were considered evaluable for adverse events (Table 2). Of the 23 patients, two patients opted to withdraw early due to intolerable adverse events which included rash and decrease in appetite in one patient and headaches in the other patient. Dose reductions and modifications were otherwise made in 7/21 (33%) patients. The most common grade 1-2 adverse events were rash (78%) and fatigue (57%). Grade 3 drug related adverse events were experienced in 13/23 (57%) patients. There were no grade 4 adverse events and no deaths due to drug-related adverse events.

**Table 2:** Toxicity (N = 23)

Drug related adverse event	Grade 1-2	Grade 3
Dermatologic		
Rash	18 (78%)	0
Pruritus	1 (4%)	0
Dry Skin	2 (9%)	0
Hand/foot syndrome	6 (26%)	1 (4%)
Photosensitivity	1 (4%)	0
Alopecia	5 (22%)	0
Flushing	3 (13%)	0
New primary melanoma	0	1 (4%)
Neurologic		
Abducens nerve disorder	1 (4%)	0
Syncope	0	1 (4%)
Neuropathy	2 (9%)	0
Photophobia	1 (4%)	0
Blurred vision	2 (9%)	0
Headache	8 (35%)	0
Gastrointestinal		
Diarrhea	4 (17%)	0
Nausea/Vomiting	4 (17%)	0
Dyspepsia	1 (4%)	0
Dysgeusia	1 (4%)	0
Anorexia	2 (9%)	0
Laboratory		
Anemia	4 (17%)	0
Hypophosphatemia	0	4 (17%)
Leukopenia	0	5 (22%)
Transaminitis	0	1 (4%)
Other		
Fatigue	13 (57%)	0
Arthalgia/myalgia	8 (35%)	0
Sore throat	1 (4%)	0
Pyrexia	7 (30%)	0
Insomnia	1 (4%)	0
Chills	4 (17%)	0

### cfDNA

**Figure 2:**
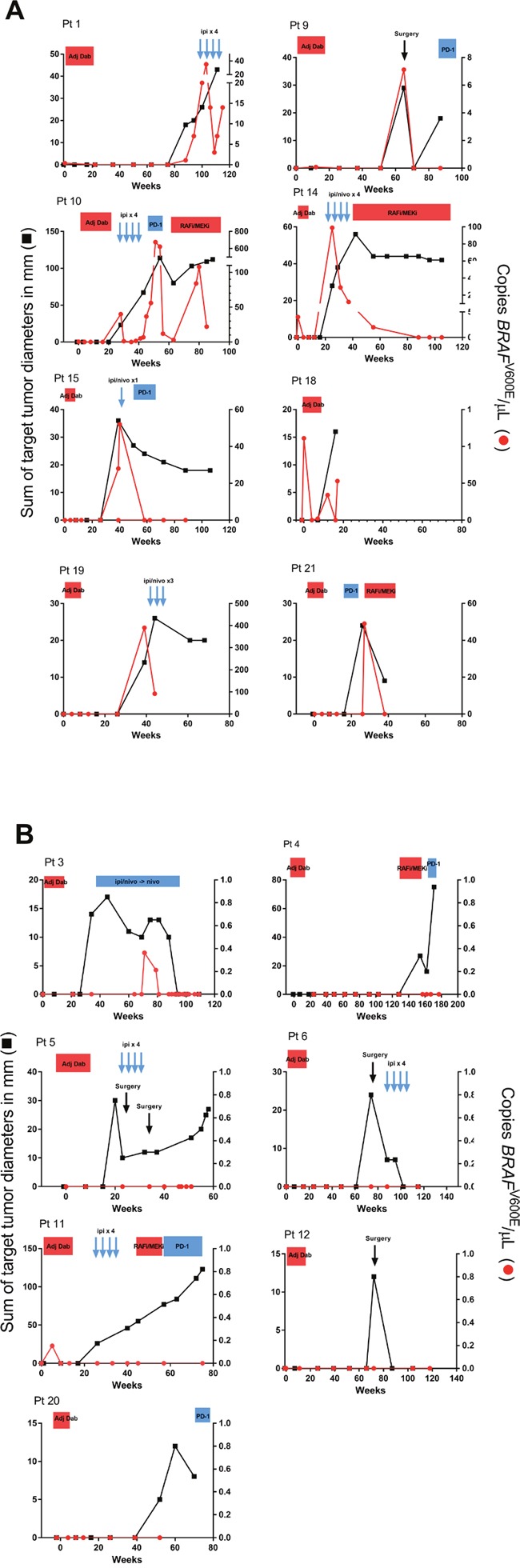
Correlation of radiographic evaluation and tumor-derived cfDNA analysis among 15 patients who relapsed **(A)** Patients in whom tumor-derived cfDNA was detected at the time of radiographic evidence of relapse. **(B)** Patients in whom tumor-derived cfDNA was not detectable at the time of radiographic relapse. Black boxes denote the sum of the target lesion diameters as measured on CT scan. Red circles indicate levels of tumor-derived cfDNA. Treatment regimens are indicated at the top of each graph. Patient #4 did not have cfDNA samples available until after completing adjuvant dabrafenib therapy. Adj Dab, adjuvant dabrafenib; Ipi, ipilimumab; RAFi, RAF inhibitor; MEK, MEK inhibitor; PD-1, anti-PD1 monoclonal antibody; Nivo, nivolumab.

Analysis of cfDNA was possible in 20 patients including 15 patients who relapsed (Figure 2). One patient (pt #8) recurred soon after starting adjuvant treatment and too few cfDNA samples were collected to make analysis meaningful. Three of 15 patients had detectable tumor-derived cfDNA pre-treatment (patients #1, #14, #18). In all 3 of these patients, the tumor-derived cfDNA became undetectable after 4 weeks of adjuvant dabrafenib.

In 8/15 relapsing patients, tumor-derived cfDNA was detected at a time point concomitant with the radiographic detection of recurrent melanoma (Figure 2A). This implies a sensitivity of 53% but also indicates that cfDNA did not provide any lead-time advantage over CT scans in detecting relapse. However, 7 patients had radiographically-detected relapse with undetectable tumor-derived cfDNA levels (Figure 2B). These represented false negatives. Among the 5 patients who remain relapse-free to date, tumor-derived cfDNA was never detected (specificity 100%).

Table 3 shows the site of first relapse in the 15 patients for whom cfDNA analysis was available. In the 3 patients whose site of recurrence was the brain, none had detectable tumor-derived cfDNA at relapse. Patient #3 developed a new NRAS -mutated primary melanoma at 3 months and required discontinuation of dabrafenib for fear of further activating the MAP kinase pathway [9]. This patient subsequently developed biopsy-confirmed metastatic disease in the spleen at 6 months. Although the splenic recurrence was found to be BRAF V600E positive, tumor-derived cfDNA at relapse was not detected (Figure 2B).

**Table 3:** BRAF cfDNA Results and Site of Relapse (N = 15)

Patient number	cfDNA positive at baseline	cfDNA positive at recurrence	Site of recurrence
1	Y	Y	Peripheral lymphadenopathy
9	N	Y	Peripheral lymphadenopathy
10	N	Y	Retroperitoneal lymphadenopathy, Brain
14	Y	Y	Porta hepatis lymphadenopathy
15	N	Y	Liver
18	Y	Y	Bone, lymphadenopathy, gall bladder
19	N	Y	Spleen, Liver
21	N	Y	Subcutaneous, intramuscular, brain
3	N	N	2^nd^ primary melanoma NRAS mutant, lung, spleen
4	N	N	Brain only
5	N	N	Brain only
6	N	N	Brain only
11	N	N	Subcutaneous, adrenal
12	N	N	Subcutaneous in-transit
20	N	N	Lung

## DISCUSSION

The majority of stage IIIC melanoma patients have micrometastatic disease at the time of resection and are at high risk of relapse. In our experience, the 2 year RFS is 24% [6]. Once metastatic disease is clinically evident, patients have many orders of magnitude more tumor cells; it has been estimated that patients with widely metastatic disease have between 10^11^-10^12^ tumor cells [10]. The primary rationale for adjuvant therapy has been that patients with micrometastatic disease have orders of magnitude fewer tumor cells and might be easier to cure [11].

We tested the RAF inhibitor dabrafenib as an adjuvant treatment in stage IIIC melanoma patients who had been rendered free of detectable disease after surgery. RAF inhibitors not only have a high rate of objective responses, but these responses mostly occur within 2 months of therapy. Therefore, we reasoned that a 4-month course of therapy would provide maximal cell kill sufficient to improve 2 year RFS. We were concerned that more prolonged therapy would be associated with increased toxicity, cost, and possibly resistance, but not with significantly more anti-melanoma effects. We are aware that more prolonged courses of adjuvant targeted therapy have been used in gastrointestinal stromal tumor (GIST) where 1 year of adjuvant imatinib was associated with improved RFS, but not OS, compared to placebo [12]. More recently, a 3-year course of adjuvant imatinib was found to be superior to 1 year of therapy for both RFS and OS [13] although the OS data require longer follow up as the curves did not separate until 3 years and there were relatively few deaths, almost half of which were not attributed to GIST. Therefore, the effect of prolonged adjuvant imatinib on GIST-specific OS remains uncertain.

Our results showed that the 2 year RFS was 28.6% leading to our inability to reject the null hypothesis. Thus, our data do not support the hypothesis that 4 months of adjuvant dabrafenib will improve RFS. There are several possible reasons for these negative results. First, it is possible that adjuvant RAF inhibition therapy improves RFS but by such a small magnitude that we could not detect it in our single arm phase II trial. It is perhaps not coincidental that the observed median 2-year RFS was 4 months longer than our expected 2-year RFS, the duration of dabrafenib therapy. This supports, but is in no way definitive, of the concept that dabrafenib functions in a cytostatic manner. One advantage of single arm adjuvant trials in homogeneous high risk patients is that it might provide an early read-out as to whether there is a signal worth pursuing in a randomized trial. To detect a smaller effect, a large randomized trial (which has already completed accrual) would be needed but the clinical value of such a small improvement in RFS in the absence of an improvement in OS would have to be weighed against toxicities and drug costs. We note that the average wholesale price of 16 weeks of dabrafenib is $46,324. Second, relapse in the brain was common. Although in patients with radiographically evident brain metastases, dabrafenib certainly breaches the blood-brain barrier and can mediate tumor shrinkage [14], it is possible that in the micrometastatic setting, the blood-brain barrier is intact preventing exposure to dabrafenib. Third, since this trial was started, it has become clear that combinations of RAF inhibitors + MEK inhibitors result in a higher response rate and better OS compared to RAF inhibitors alone. It is possible that combination adjuvant therapy with a RAF inhibitor + a MEK inhibitor would be superior to observation. However, the magnitude of the benefit in RFS and OS would have to be weighed against the risks. A fourth possibility for these negative results is that there may be mechanistic limitations to ERK pathway inhibition in the adjuvant setting. Suppression of ERK activation by RAF inhibitor in a BRAF V600-mutated melanoma leads to relief of negative feedback upstream of RAF and re-activation of RAS [15, 16]. This leads to low-level re-activation of the ERK pathway and presumably allows survival of a fraction of slow-cycling cells. It may be that in this way, micrometastatic melanoma cells are able to survive RAF inhibition. If this is correct, adjuvant RAF inhibitors will not eliminate all melanoma cells.

Despite a 2 year RFS rate of only 28.6%, the estimated 2-year OS was 77.5% and the median OS has not been reached. This is markedly better than our historical experience [6] or pooled international data from before the availability of effective therapy for metastatic melanoma [17] and is likely due to subsequent therapies with checkpoint inhibitors and RAF kinase inhibitors that our patients received at relapse (Table 1). We were interested to note that of the 6 patients who received RAF or MEK inhibitors in the metastatic setting, 5 responded to treatment suggesting that tumors remained sensitive to RAF inhibition despite the short course of adjuvant dabrafenib. This is consistent with a recently-published phase II trial in which 8 of 25 patients rechallenged with dabrafenib + trametinib responded after having previously progressed and been taken off BRAF inhibitor therapy [8]. In our one relapsing patient who did not respond to RAF inhibition, the recurrent melanoma was found to be BRAF WT by next-generation sequencing. This would explain the lack of response. It is unlikely that this represented a subclone of the original melanoma but could represent a second melanoma. We cannot rule out the possibility that the determination of BRAF mutation in the original melanoma (which had been tested by immunohistochemistry) had been a false positive.

We serially measured circulating tumor-derived cfDNA using a sensitive digital PCR method and compared this with cross-sectional imaging. This allowed a prospective comparison of our cfDNA assay with standard radiographic evaluation to detect recurrence. Although we analyzed only 15 patients, this represents one of the largest prospective cohorts in which radiographic assessment was done concomitantly. Others have reported experiences using similar techniques but collected samples from small numbers of patients not on standard treatment nor using a standardized radiographic assessment schedule [18–20]. We found that measuring circulating tumor-derived cfDNA was less sensitive than CT scans in detecting recurrent melanoma. Seven of 15 patients with recurrences did not have detectable tumor-derived cfDNA at the time of recurrence which yielded only a 53% sensitivity. Of the patients with detectible tumor-derived cfDNA at recurrence, there was little lead-time advantage in measuring cfDNA, although it was interesting that 3 patients had detectable tumor-derived cfDNA at the time of starting adjuvant dabrafenib. Although there were no false positive cfDNA results among the 5 patients who never recurred, we find that tumor-derived cfDNA detection was insufficiently sensitive to replace standard cross-sectional imaging for detection of recurrent melanoma. The 3 patients who recurred only in the brain were among the false negative patients and raises the suggestion that cfDNA from the CNS may not cross the blood-brain barrier efficiently.

To date, the only FDA approved adjuvant systemic therapies for melanoma are high dose interferon alfa and high dose ipilimumab, although FDA approval of adjuvant ipilimumab occurred well after our trial had completed accrual. Randomized studies with interferon have shown a modest RFS benefit but no significant consistent OS benefit [21, 22]. Adjuvant ipilimumab (10mg/kg) was associated with improvement in RFS compared to placebo (median RFS of 26.1m vs 17.1m in favor of ipilimumab) [23, 24]. There was also an 11% improvement in 5 year OS (65.4% vs. 54.4%). However, interpretation of these data is tempered by the fact that for most patients in the placebo group who progressed, checkpoint inhibitor therapy was not available. It remains an open question, therefore, whether adjuvant therapy that has a small effect on RFS offers survival advantage over treating with anti-PD1 based checkpoint inhibition therapy at the time of relapse.

Our trial is limited by its small size and we await results of large randomized adjuvant phase III trials with RAF +/− MEK inhibitors. Our data predict that the effect on RFS will be small. If there is a difference in RFS, we will have to weigh this against the toxicities and financial costs. A critical question will be whether there is a detectable OS advantage for adjuvant therapy given that the tumors seem to remain sensitive to BRAF inhibition and effective treatment with checkpoint inhibitors are available.

## MATERIALS AND METHODS

### Patients

Eligible patients were at least 16 years old and had undergone complete surgical resection of AJCC (v. 7) stage IIIC melanoma within 90 days. Histologic confirmation of melanoma was performed in the Department of Pathology, Memorial Sloan Kettering Cancer Center. The melanoma had to harbor either a BRAF V600E or BRAF V600K mutation which was documented either by genotyping [25] or immunohistochemistry [26] performed by a CLIA certified laboratory.

Patients were permitted to have adjuvant radiation therapy but all treatment had to have been completed and patients adequately recovered before study start. Brain imaging (CT scan or MRI) and ECG were required within 4 weeks of study start and radiographic assessment of the chest, abdomen and pelvis was required to assure no evidence of distant disease within 2 weeks of study start.

Important exclusion criteria included an Eastern Cooperative Oncology Group (ECOG) performance-status score of ≥2, a prior history of stage IIIA or IIIB melanoma that subsequently progressed to stage IIIC, prior systemic adjuvant therapy for melanoma, a concurrent second malignancy, or a QTc interval >500 msec unless a bundle branch block was also present.

### Study design, regimen, and assessments

This study was a single institution, single arm, phase II trial. The trial was approved by the institutional review board and patients were consented and registered at Memorial Sloan Kettering Cancer Center. All eligible patients received adjuvant dabrafenib at a dose of 150 mg twice daily by mouth for 4 cycles. One cycle was defined as 28 days (4 weeks). The primary endpoint was recurrence-free survival at 24 months. The secondary endpoints included overall survival and toxicity.

All patients were evaluated at the start of each 4 week cycle +/− 5 days with physical examination, standard and correlative laboratory studies. Radiographic evaluation of disease status was obtained at the end of cycles 2 and 4. Dermatologic exams were performed at the completion of cycles 2 and 4 and ophthalmologic exams were performed at the completion of cycles 1 and 3. Dabrafenib was discontinued after 4 cycles of adjuvant therapy, or sooner for disease recurrence or toxicity that did not improve with the recommended dose reductions as outlined in the full trial protocol.

After the completion of adjuvant dabrafenib, patients were evaluated every 3 months +/− 2 weeks with physical examination, standard blood tests, blood draws for cfDNA (see below), and radiologic evaluation of disease status. This assessment was repeated every 3 months until the completion of active follow-up at 24 months from the date of surgical resection at recurrence. All recurrences were confirmed by biopsy and histologic evaluation.

Adverse events were graded based on the Common Terminology Criteria for Adverse Events (CTCAE) Version 4.0. Adverse event evaluation was performed on all patients who received at least one dose of dabrafenib.

### cfDNA

We made serial measurements of tumor-derived cfDNA in the plasma to correlate with disease status as assessed by standard cross-sectional imaging. Patients underwent serial peripheral blood draws pretreatment and at each radiographic evaluation. The peripheral blood was collected in cell-free DNA BCT tubes (Streck, Inc) although in some of the early patients, EDTA tubes were used. The tubes were centrifuged and processed within 2 hours of collection. The blood was centrifuged at 820 x g for 10 minutes, and then the plasma was transferred to sterile Eppendorf tubes in 1cc aliquots and centrifuged at 16,000 x g for 10 minutes. The supernatant was transferred into cryovials in 1cc aliquots and stored at −20C. cfDNA extraction was initially accomplished using Qiagen QiAamp Circulating Nucleic Acid Kits according to the manufacturer's instructions and the concentration was assessed using BioAnalyzer (100-300nt). Later in the study, cfDNA was isolated using QIAsymphony DSP virus/pathogen midi kits according to the manufacturer's instructions. cfDNA was isolated from 3 ml of plasma. The automated extraction process included sample lysis and binding to magnetic particles (3x lysis and binding) using the Qiagen MagAttract “G” particles (ferromagnetic particles with a mean diameter of 6-10 μm). The sample was then washed and eluted. cfDNA was quantified either using the 2200 TapeStation instrument (Agilent Technologies, high sensitivity D1000 screen tape and reagents) or Fragment Analyzer (Advanced Analytical, high sensitivity genomic kit). The TapeStation software calculates the concentration of cfDNA. Tumor-derived cfDNA was quantitated by digital droplet PCR using probes for BRAF V600E (1799T>A) mutation and BRAF V600K (1798_1799GT>AG) mutations (BioRad QX200 Hercules, CA) essentially as previously described. This assay can detect reliably 5 molecules of BRAF V600E DNA fragments/ml blood [27].

### Statistical analysis

The primary endpoint of the trial was the 24 month relapse-free survival rate. Relapse-free survival was defined as the time from surgical resection to the first recurrence or death as assessed by physical examination and radiographic evaluation. Overall survival was a secondary endpoint and defined as the time from surgical resection to death or last follow-up.

A 24% relapse-free rate at 24 months was considered not promising while a 51% relapse-free rate at 24 months was considered promising. The probabilities of a type I error (falsely accepting a non-promising therapy) and type II error (falsely rejecting a promising therapy) were each set at 0.10 and 0.10. This design yields a 0.90 probability of a positive result if the true relapse-free survival is at least 51% and yields a 0.90 probability of a negative result if the true relapse-free survival is 24%. At the end of the trial, if 9 or more patients out of 23 patients were relapse-free at 24 months, we would be able to reject the null hypothesis indicating that adjuvant dabrafenib would be worthy of further investigation. The trial would be terminated early if at any time 15 patients relapsed or died before 24 months.

All patients were included in the intention-to-treat analysis except for those patients who received less than 1 week of therapy and elected to withdraw consent.
